# Recombinant *Mycobacterium bovis* BCG-Based HIV Vaccine: Failures and Promising Approaches for a Successful Vaccine Strategy

**DOI:** 10.3390/vaccines13060606

**Published:** 2025-06-03

**Authors:** Joan Joseph-Munné, Milena Maya-Hoyos, Narcís Saubi, Santiago Perez, Miguel Angel Martinez Lopez, Eder Baron, Carlos Yesid Soto

**Affiliations:** 1 Microbiology Department, Vall d’Hebron University Hospital, Vall d’Hebron Research Institute (VHIR), 08035 Barcelona, Spain; narcis.saubi@vhir.org (N.S.); eder.baron@vhir.org (E.B.); 2 Chemistry Department, Faculty of Sciences, Universidad Nacional de Colombia, Bogotá 111321, Colombia; mmayah@unal.edu.co; 3 Microbiology Department, Hospital de Mollet, Fundació Sanitaria Mollet, 08100 Barcelona, Spain; mig.martinez@fsm.cat

**Keywords:** HIV, rBCG, BCG, AIDS, live-attenuated vaccines, mycobacteria

## Abstract

During 2022, AIDS claimed a life every minute and about 9.2 million HIV-infected people were not on treatment. In addition, a person living with HIV is estimated to be 20–30 times more susceptible to developing active tuberculosis. Every year, 130,000 infants are newly infected, with vertical transmission being the main cause of pediatric HIV infection. Thus, the development of an effective, safe, and accessible vaccine for neonates and/or adults is an urgent need to prevent or control HIV infection or progression to AIDS. An effective HIV vaccine should induce long-lasting mucosal immunity, broadly neutralizing antibodies, innate immunity, and robust stimulation of CD4+ and CD8+ T-cell responses. Recombinant BCG is a promising live-attenuated bacterial vaccine vector because of its capacity to stimulate T-cell immunity. As a slow-growing microorganism, it provides prolonged low-level antigenic exposure upon infecting macrophages and APCs, potentially stimulating both effector and memory T-cell responses. BCG is considered safe and is currently administered to 80% of infants in countries where it is part of the national immunization program. Additionally, BCG offers several benefits as a live vaccine vehicle since it is cost-effective, easy to mass-produce, and heat stable. It is also well-suited for newborns, as maternal antibodies do not interfere with its efficacy. Furthermore, BCG has a strong safety profile, having been administered to over three billion people as a TB vaccine. In this review, we provide an extensive summary of the literature relating to immunogenicity studies in animal models performed since 2011. Moreover, we provide a comprehensive analysis of the key factors influencing the design of recombinant BCG as a live vaccine vehicle: (i) expression vectors; (ii) selection of HIV immunogen; (iii) promoters to regulate gene expression; (iv) BCG strain and BCG codon optimization; (v) genetic plasmid stability; (vi) influence of preexisting immunity, route, and dose immunization; and (vii) safety profile.

## 1. Introduction

Globally, about 39% fewer people acquired the human immunodeficiency virus (HIV) in 2023 compared to 2010, with sub-Saharan Africa achieving the steepest reduction (−56%). Nonetheless, an estimated 1.3 million [1.0 million–1.7 million] people acquired HIV in 2023, which is over three times more than the target of 370,000 or fewer new infections in 2025 [1]. Over the last thirty years, increased access to HIV treatment has prevented nearly 20.8 million AIDS-related deaths. However, although progress has been made, in 2022, AIDS claimed a life every minute [2]. Notably, about 9.2 million people living with HIV were not receiving treatment, and approximately 2.1 million were on treatment but not virally suppressed. In addition, a person living with HIV is estimated to be 20 to 30 times more susceptible to developing active tuberculosis. The progress in combating AIDS represents a significant public health accomplishment, especially given the lack of a vaccine for prevention or a definitive cure. However, in a world marked by intersecting inequalities, not everyone is benefiting yet [2]. Even though highly effective strategies to eradicate HIV vertical transmission have averted 3.4 million infections in children, protective programs have only reached 53% of pregnant and breastfeeding women in western and central Africa [2]. Every year, 130,000 infants are newly infected, with vertical transmission being the main cause of pediatric HIV infection [3,4].

T-cell mediated immunity plays a crucial role in controlling HIV replication and may be key for HIV protection [5,6]. An effective HIV vaccine should induce long-lasting mucosal immunity, broadly neutralizing antibodies, innate immunity, and robust stimulation of CD4+ and CD8+ T-cell responses. Furthermore, HIV vaccines must be safe, stable, affordable, and easy to store without the need of a cold chain as they are in need in resource-poor settings with a high prevalence and incidence of HIV and TB infection.

*Mycobacterium bovis* bacillus Calmette–Guérin (BCG) is the current licensed vaccine for tuberculosis (TB), which protects against childhood TB meningitis, milliary TB disease, and leprosy, and is currently administered to 80% of infants in countries where it is part of the national childhood immunization program [7]. Subsequent worldwide distribution of BCG and repeated subcultures have led to a loss of virulence in BCG [8]. The most relevant advantages of BCG as a live-attenuated vaccine vector are: (1) it is easy to mass-produce at low cost; (2) it is heat stable; (3) it has a long record safety profile, with= over 3 million individuals immunized; (4) it is suitable for neonates as vaccination is not affected by maternal antibodies; and (5) it acts as an adjuvant on its own due to its cell-wall components and lipoproteins.

BCG is a promising live-attenuated vaccine vehicle for inducing specific humoral and long-lasting innate, CD4+, and CD8+ T-cell responses against HIV-1 following recombinant BCG (rBCG) expressing HIV-1 antigens [9,10,11,12,13,14]. In addition, a variety of viral, bacterial, parasitic, and human antigens have been successfully expressed in BCG. In experimental models, rBCG has elicited protective immunity against measles, papillomavirus, listeriosis, malaria parasites, pneumococcal infection, pertussis, Lyme disease, leishmaniasis, and others [15,16,17,18,19,20,21,22].

rBCG expressing HIV immunogens have been assessed in animal models with encouraging results, a full description of which is given in this review. However, to achieve robust immunogenicity and protection against HIV using this mycobacterial vaccine design, it is crucial to carefully consider and design genetic tools for stable expression of HIV antigens in BCG. There are several determinant factors that are critical issues for the rational design and evaluation of rBCG:HIV vaccine candidates. These include, among others: (1) the influence of age, dose, and immunization routes (2) the selected HIV antigen; (3) the type of expression vectors (replicative or integrative) used to express the recombinant proteins and ensure the genetic stability of the vector in vivo; (4) the strength of the promoter selected for the HIV gene expression regulation; (5) the BCG strain used as a carrier; (6) the preclinical animal models selected to test the recombinant strains; and (7) the use of secretion signals to increase the presentation of peptide antigens on major histocompatibility complex (MHC) classes I and II. These features are reviewed in this paper.

## 2. Determinant Factors Involved in the Design of Recombinant BCG as a Vaccine Vehicle

BCG vaccine is a live-attenuated bacterial vaccine derived from *Mycobacterium bovis,* and it is the only vaccine against TB currently in use. It was originally isolated in 1908 from a cow with TB. The isolate was cultured by serial passage for 13 years (from 1908 to 1921) to generate a mutant strain with low virulence but high immunogenicity [23,24]. BCG was first used as a vaccine in 1921 when it was administered to a boy, and has been part of the World Health Organization (WHO)’s infant immunization programs since 1974 [24]. The effectiveness of the BCG vaccine varies depending on various factors, such as the strain used, the age at vaccination, and the prevalence of TB in each population. BCG provides significant protection against disseminated and meningeal TB in pediatric populations (lasting up to 10 years), but shows variable efficacy against pulmonary TB in adults and adolescents [25].

There are well-recognized genomic and proteomic differences between BCG and the *M. tuberculosis* (*Mtb*) complex species. Specifically, the *Mtb* and *M. bovis* genomes have similar sizes (~4.4 to 4.5 million bp) and genomic organization. However, there are notable differences due to evolutionary divergence and horizontal gene transfer events, including genes encoding virulence factors, metabolic enzymes, and cell-wall components [26]. For example, nine open reading frames within the region of difference 1 (RD1) locus of *Mtb* containing genes coding the TB-specific immunodominant antigens ESAT-6 and CFP-10 proteins are absent in the BCG genome [27]. Thus, this BCG attenuation would be of interest in the development of new vaccine candidates against TB. In addition, heterologous expression of these and other antigens, such as Ag85B and the mycobacterial ESX-1 secretion system, are used to enhance the BCG cellular immune response. Concerning the COVID-19 pandemic, some studies have suggested an inverse correlation between BCG vaccination coverage and lower morbidity and mortality rates from COVID-19, which could be due to prior exposure to the TB bacillus and the mortality-reducing effects of the BCG vaccine, especially in countries with universal BCG vaccination policies [28,29]. In addition, BCG vaccination policies vary widely between countries, so public health interventions can influence COVID-19 outcomes. Therefore, *Mtb* and *M. bovis* exhibit genomic and proteomic differences that contribute to their unique characteristics as pathogens. They must be understood to develop effective diagnosis, treatment, and prevention strategies.

### 2.1. Escherichia coli-Mycobacterium Shuttle Vectors

Gene expression requires successful molecular microbiology strategies and studies at the cellular level. Usually, *Escherichia coli* (*E. coli*) is an extensively used bacterial system for protein expression, but large mycobacterial proteins are not adequately expressed in *E. coli* due to the formation of insoluble inclusion bodies [30,31]. The study of mycobacteria using molecular genetic techniques involves the insertion of recombinant DNA into mycobacterial cells. For this reason, the *E. coli-Mycobacterium* shuttle vector systems have been developed, which are multifunctional as they allow for replication in either *E. coli* or mycobacterial strains [30,31]. The *E. coli-Mycobacterium* shuttle vector can be transformed into a wide variety of mycobacterial species, including *M. smegmatis*, *Mtb,* and *M. bovis* BCG, and have been widely used for homologous or heterologous expression of proteins in mycobacteria [31,32].

In the first attempts, the shuttle vectors were a collection of *E. coli* cosmids (such as pHC79) inserted at random sites into the nonessential region of temperate mycobacteriophages such as TM4, L1, or D29. These shuttle phasmids were packaged into bacteriophage/mycobacteriophage particles. Therefore, they replicated as plasmids in *E. coli* and as phage in mycobacteria [32]. However, these shuttle phasmids were difficult to manipulate because they required viral particles for packaging and transfection, and were sometimes shown to be lytic for *M. smegmatis* and BCG [32,33,34]. Then, a new generation of *E. coli*/*Mycobacterium* shuttle vectors, such as p15A, ColE1, and pBR322, for heterologous expression of proteins in mycobacteria were developed based on the *M. fortuitum* plasmid pAL5000 and the *E. coli* origin of replication (ori*M* and ori*E*), respectively [35]. This allowed for= recombinant selection in both *E. coli* and mycobacterial species [36,37]. Indeed, the pJEM vectors derived from pAL500 allowed for the detection of mycobacterial promoters and the identification of sequences involved in mycobacterial protein secretion [36]. Other pAL5000-derived vectors include the pSUM family (containing pACYC184 *E coli* plasmid and kanamycin-resistance *aph* gene) [36] and pAL8 (containing pTZ19R *E. coli* plasmid and kanamycin-resistance gene) [38], among others.

Remarkably, BCG can be used as an expression vector for bacterial and viral antigens to develop a rBCG as a promising recombinant vaccine [37,39,40]. Although BCG protects against TB by inducing both humoral and cellular immune responses, diverse studies have shown that BCG induces non-specific effects (NSEs) that provide effective protection against other infectious diseases [24], such as a respiratory syncytial virus [41], human papillomavirus [42], and herpes simplex virus [43], among others. BCG is a potent adjuvant that enhances immunogenicity against foreign antigens expressed in rBCG [44] and can induce non-specific cross-protection against non-target pathogens through heterologous T-cell immunity and trained immunity conferring non-specific immune memory [24,39].

Moreover, high levels of protein expression using replicative *E. coli-Mycobacterium* shuttle vectors have been associated with genetic instability in rBCG [37,45], caused by high copy numbers, constitutive promoters, or protein toxicity [37,46]. In this context, several alternative expression systems have been developed that allow for protein expression under the control of constitutive promoters (such as *Ag85* [47] and *rel* [48]) or inducible promoters (such as heat shock protein of 60 kDa (*hsp60*) [35], tetracycline [49], and acetamide [31,50]), as described below. Integration signals of mycobacteriophages have been key to the development of site-specific integrative vectors. Some of them derived from temperate mycobacteriophages (L5 and Ms6) show greater stability because they integrate as a single copy into the mycobacterial genome [51,52]. Therefore, these plasmids have been an excellent alternative for heterologous gene expression and bactofection in mycobacteria. However, alternative genetic methods are required to overcome the limitations of mycobacterial recombinant systems [37].

#### 2.1.1. Replicative Vectors

The replicative *E. coli*/*Mycobacterium* shuttle vectors are maintained in mycobacteria in a multicopy and extrachromosomal manner [37]. As mentioned above, most of the mycobacterial replicative vectors for foreign antigens expression in BCG are derived from the *M. fortuitum* pAL5000 plasmid. These vectors allow about five copies of the plasmid per cell, resulting in higher levels of recombinant proteins [44]. In some cases, the high levels of recombinant antigens expressed in BCG impose a metabolic burden and possible toxicity for mycobacteria [53]. High protein levels can cause stress in BCG and the induction of the mechanism to counteract the deleterious effects of protein overexpression [54]. Thus, reduced expression of heterologous antigens may be compensated by sustained synthesis of the foreign antigen and induction of long-lasting immunity [44,55].

The pYUB family was the first generation of replicative shuttle vectors for mycobacteria. They were constructed from the pIJ666 *E. coli* and pAL5000 plasmids, which contain the *hps*70 promotor and neomycin-kanamycin/chloramphenicol resistance, allowing for selection in both *E. coli* and BCG [33,56]. Specifically, the pYUB12 plasmid was used to express fragments of the HIV-1 *gag*, *pol*, and *env* genes in BCG, which elicited antibodies to HIV-1 and cytotoxic T-cell responses in mice [57].

As mentioned above, the replicative shuttle vector pMV261 has been widely used for protein expression in mycobacteria. It contains an *E. coli* plasmid replicon derived from pUC19 (ori*E*), a mycobacterial DNA origin of replication (ori*M*), a kanamycin-resistance *aph* gene, the *hsp60* mycobacterial promoter, a multiple cloning site, and a transcriptional terminator. pMV261 allows for high transformation efficiencies of BCG and maintains approximately five plasmid copies per genome [35]. Some studies have demonstrated in vitro instability of pMV261 when the gene expression was upregulated by a strong promoter [58,59].

Another commonly used shuttle vector is pJH222 (derivate from pMV261), a low-copy-number replicative vector that contains ori*E* (from *E. coli*), ori*M* (from *M. fortuitum*), the *Mtb* α-antigen promoter, a multiple cloning site, a transcriptional terminator, and a wild-type *lysine A* complementing gene (under the control of the BCG *hsp60* promoter) for vector maintenance in the BCGΔ*lys*A mutant strains [59]. pJH222 was used to express the HIV-1 clade A-derived immunogen (HIVA). The HIVA immunogen consists of consensus HIV-1 clade A Gag p24/p17 domains coupled to a sequence of CD8+ T-cell epitopes and the monoclonal antibody (mAb) tag Pk [60,61]. BCG.HIVA prime (rBCG with the episomal vector pJH222.HIVA), in combination with modified vaccinia virus Ankara MVA.HIVA, boost induced durable HIV-1-specific CD4+ and CD8+ T -cell and *Mtb*-specific responses in BALB/c mice. They also protected against surrogate virus challenge, while the recombinant BCG vaccine alone protected against the aerosol challenge with *Mtb* [39,62,63].

Another replicative vector is pMyong2, an 18-kb linear plasmid from *Mycobacterium yongonense* DSM 45126 a complement to the conventional pAL5000-derived vector [37]. The pMyong2 vector allowed 37 times more plasmid copies, and 50-fold higher expression levels than the pAL5000 plasmid [37,53]. The pMyong2 vector was used to express HIV-1 Gag p24 antigens in *M. smegmatis* (rSmeg-pMyong2-p24) and to evaluate cellular and humoral immune responses against HIV Gag proteins in vaccinated mice compared to rSmeg strains transfected with pMV306 an integrative plasmid [64]. The mice vaccinated with rSmeg-pMyong2-p24 elicited more effective immunity compared to the counterpart integrative rSmeg-pV306-p24, as evidenced by higher levels of HIV-1 Gag-specific CD4+ and CD8+ T lymphocyte proliferation, INF-γ induction, and antibody production. These results suggest that pMyong2 is an alternative *E. coli*/*Mycobacterium* shuttle vector system for antigen expressing in vaccine applications [64].

A lysine auxotroph of the vaccine MTBVAC (MTBVACΔ*lys*) transformed with the *E. coli*-*Mycobacterium* vector p2Auxo.HIVA (episomal, replicative), which contains the *lysA*-complementing gene and the immunogen HIVA, was used as a vector for a dual-TB–HIV vaccine as one of the first attempts at TB–HIV vaccines [65]. This dual vaccine, combined with MVA.HIVA, showed similar efficacy to mice vaccinated with the parental strain and challenged with *Mtb*. It was also safer than BCG in a SCID mouse model, induced HIV-1 and *Mtb*-specific IFN-γ producing T-cell responses, and HIV-1-specific CD8+ T-cell producing IFN-γ [65].

#### 2.1.2. Integrative Vectors

The expression levels of foreign genes are high using replicative vectors, but the genetic stability is poor, which may affect the induction of immune responses [53]. Integrative vectors are more stable in vitro and in vivo than replicative vectors in BCG [44,55,66]. The integrative *E. coli*/*Mycobacterium* shuttle vectors have been developed to insert a single gene copy via integration into the mycobacterial chromosome and have been a good alternative for the expression of foreign antigens in BCG [37,67].

The integration event prevents mutation of the integrated gene in mycobacteria due to its location within the genomic DNA [34,53]. Integrative vectors are usually maintained in a single copy into the mycobacterial genome, allowing for lower expression levels and metabolic load compared to replicative vectors [44,53]. Thus, a reduced metabolic burden allows for more stability in vivo [53]. The use of integrated vectors avoids the risk of horizontal vector transfer with other pathogens, which improves the safety of recombinant vaccines [53]. The long-lasting stable expression of heterologous antigens using integrative vectors achieves a long-lasting immune response [44,53].

In general, an integrative vector carries a mycobacteriophage integrase gene (*int*) and phage attachment site (*attP*) from mycobacteriophages (such as L5 or Ms6) that is homologous to the bacterial attachment site *attB* (located within the *glyV* tRNA gene) [52,68]. The integration systems use the L1 mycobacteriophage genes in the phAE19 vector series [33], Ms6 in the pEA4 series [69], and the L5 in the pMH94 [52] and pMV361 series [35]. The phage integrase (*Int*) with the mycobacterial integration host factor (mIHF) produces a recombination event between the *attP* and *attB* sites of the mycobacterial genome, resulting in the integration of a sequence of vectors into the bacterial chromosome [52,68]. Alternatively, the integrative vectors can be removed from the mycobacterial genome using the mycobacteriophage L5 excisionase (Xis) and the Int proteins previously contained in the recombinant plasmids [34,70]. In addition, there are integrative plasmids containing the bacterial *attB* site, which provide an additional copy of this integration site to the bacterial genome after the integration event. This additional *attB* site allows for the integration of another integrative plasmid [34,68,71].

The integration-proficient pMV361 vector was constructed by replacing the Ori*M* of pMV261 with a DNA sequence carrying the *attP site* and *int* gene from the mycobacteriophage L5 [35]. BCG transformed with pMV361, a site-specific integration into chromosomal *attB* site, showed similar efficiency to that of extrachromosomal vectors [35]. Another commonly used mycobacterial integrative vector is pJH223, derived from the pJH222 replicative vector, which contains a kanamycin-resistance gene, Ori*E*, the *lysA*-complementing gene under the regulatory control of the *hsp60* promoter, a DNA segment carrying the *attP site* and *int* gene from mycobacteriophage L5, and an expression cassette containing a mycobacterial promoter (α-antigen *Mtb* promoter), a multiple cloning site and a transcriptional terminator [59]. Additionally, the recent integrative vector used is p2auxo^int^, an antibiotic markerless vector that contains ori*E*, the *attP* site*,* and the *int* gene from mycobacteriophage L5, as well as the *glyA* and *lysA* genes for maintenance in *E. coli* and BCG auxotrophic strains, respectively [72].

### 2.2. Promoters to Regulate Gene Expression

The selection of suitable promoters is essential to achieve optimal expression levels of homologous or heterologous proteins in rBCG [73]. Promoters in mycobacteria are generally located in the intergenic regions; according to their functioning, type, and expression level, they are classified into: (i) constitutive promoters expressed only by their interaction with the RNA polymerase, which display high transcription levels; (ii) inducible promoters activated by different stimuli, such as chemical and physical signals, or under controlled laboratory conditions; and (iii) Synthetic or chimeric promoters, where the consensus sequence, copy numbers, and distance to the transcription start point (TSP) have been rearranged, allowing for coordinated control of multiple genes [74]. The hexamer −10 sequence (TATA box) from the TSP (+1) is similar between *E. coli and* mycobacteria. Conversely, the −35 sequence varies considerably in mycobacteria, mainly due to differences in the codon frequency and the multiplicity of sigma factors (σ) [75]; that is, there are at least 13 sigma factors in the *Mtb* genome [26]. Unlike the TATA box, the −35 regulatory region is not essential for gene transcription in mycobacteria [73].

Classical promoters for the expression of mycobacterial proteins, such as *hsp60*, are useful if only high transcription levels are needed. The *hsp60* promoter is induced under stress conditions, such as temperature and intracellular growth [35]. However, protein expression under this strong promoter is unstable; rearrangements and insertions/deletions of its nucleotide sequence are common, and it is also not recommended for expression of toxic proteins [54,59,67]. The plasmid pMV261 (under the *hsp60* control) is one of the most widely used vectors for the expression of mycobacterial proteins in *M. smegmatis*, *M. bovis*, and *Mtb* [34]. Sometimes this vector does not display adequate levels of the expressed protein. In a recent study of infection in a murine alveolar macrophage cell line (MH-S), complementation of an *Mtb* strain (*Mtb*Δ*ctpF*), defective in the plasma membrane Ca^2+^ transporter CtpF associated with virulence, was deficient when using pMV261 as a replicative expression vector. The complemented strain (*Mtb*∆*ctpF*:*ctpF*) exhibited a 75-fold reduction in *ctpF* replicative capacity and impaired virulence compared to the wild-type strain (*Mtb*H37Rv) during the first 7 days after infection [76]. In this case, multicopy vectors with strong promoters (*hsp60*), such as pMV261, produce high levels of recombinant proteins, which could be toxic to mycobacteria [54,67]. An alternative for working with *Mtb* defective mutants in cellular or animal infection models is to use moderate expression of complementing proteins mediated by the *hsp70* promoter. In this case, the promoter is induced with H_2_O_2_, which, in turn, is increased under infection conditions. Protein expression under the *hsp70* promoter has been useful for different mycobacteria, especially in *M. smegmatis* [77].

On the other hand, the expression stability of recombinant proteins in mycobacteria can be improved by using integrative expression vectors. With this type of vectors, the promoter strength is controlled, generating less metabolic burden on the host [34,61]. This is the case with integrative vectors such as pMV361, in which the *hsp60* promoter is integrated together with the expressed protein in the mycobacterial genome and the frequency of mutations diminishes by 1000-fold compared to the counterpart replicative vector pMV261 [66]. Differences were also observed when using the replicative or integrative *hsp60* promoter for expressing the VP6 protein in rBCG for vaccine purposes against rotavirus [54]. In this case, the protein expression under the control of the integrated promoter was considerably reduced, diminishing the bacterial toxicity. Similarly, controlled protein expression at wild-type levels can be obtained using vectors under the control of the secreted 85 antigen (Ag85) promoter. Indeed, constitutive complementation of *Mtb DosR* null-mutants in a mouse model using the integrative plasmid pEM37 was possible in the mid-exponential phase, recovering the attenuation phenotype. This expression system is thus recommended to complement *Mtb* or BCG mutants for genes located in the middle of an operon [78].

There are also alternative promoters that have been used to regulate the gene expression of heterologous proteins in rBCG. The *M. leprae* 18 KDa promoter shows 0.8–2.8-fold higher protein expression levels under in vitro hypoxia and starvation compared with aerobic conditions [79]. Among alternative promoters, those with the possibility of controlled induction are also especially useful for studying the phenotypic behavior of toxic proteins [80]. One of the first described and most commonly used inducible promoters is the *M. smegmatis amiE*, which is induced by acetamide, as a primary carbon source [81]. *amiE* functions best in *M. smegmatis*; however, it shows instability for protein expression in *Mtb* [82]. Similarly, the *hspX* promoter of the *Mtb* chaperone HspX (α-crystalline) is useful for studies of latency that involve hypoxic conditions, especially in in vitro infection models [83]. *hspX* has also shown augmented expression of proteins in BCG during cellular infection models displaying adequate T-cell priming and IFN-γ production [84]. Otherwise, there are induced expression promoters that are inefficient in mycobacteria. This is the case with P_BAD_ induced with arabinose [85] and the TetON system inducible by tetracycline [49]. The latter requires codon optimization since it originates from *E. coli* and is not recommended for cellular infection studies [86]. Regarding the expression of HIV antigens, the promoter of the mycobacterial signal transducer system MrtA [87] shows stability in rBCG autotrophs expressing HIV Gag proteins for vaccine purposes [88]. Similarly, moderate levels of the HIV-1 gp120 protein expression are obtained using the weak α-antigen promoter in rBCG, avoiding the genomic instability typical of strong promoters such as *hsp60* [59].

Therefore, factors such as the genetic manipulation process, the selection pressure exerted by the host immune system, and the expression levels of antigens expressed in rBCG mediated by the promoter/transcriptional activity may all combine to produce genetic stability/instability. In addition to genomic rearrangements, deletions or mutations within the promoter sequence, and possible toxic effects due to high levels of expression in episomal systems [53], it is also possible that rBCG strains are under selective pressure from the immune system due to oxidative and nitrosative stress. This leads to evasion of immunological recognition or, in the case of integrative vectors, reversion to the wild-type phenotype by homologous recombination. Therefore, if a certain level of antigen expression is required in rBCG, a delicate balance must be considered between the strength of the promoter, the type of vector (integrative or episomal), and the location and sequence of the antigen to be exposed to the immune system, as will be discussed below.

### 2.3. Transcriptional Termination Mechanisms

Mechanisms of transcription termination in bacteria, including mycobacteria, are indispensable for the accurate release of RNA polymerase after RNA synthesis. There are two main bacterial termination mechanisms: (i) intrinsic termination, which involves palindromic sequences downstream of the stop codon; these are GC-rich RNA hairpins followed by 7-to-9 nucleotides uridines (U-tract) transcribed into RNA, which promote the separation of the DNA–RNA hybrid (elongation complex) in the transcription termination [89] and, (ii) those mediated by the ATP-dependent translocase/helicase Rho protein, in which the protein directly cleaves nascent RNA from RNA polymerase [90].

Correct termination of transcription in mycobacteria, and other bacterial systems, prevents incorrect transcription of downstream genes and interference by antisense sequences. Whatever the mechanism, the presence of the transcription factors NusA and NusG is key in transcription termination [90]. The canonical GC-rich sequences and U-tracts that are common in bacterial systems are poorly represented or absent in mycobacteria [91]. Thus, the mycobacterial elongation complex (EC) is separated within a non-canonical intrinsic terminator lacking U-tracts. Therefore, mycobacterial terminators may contain modified U-tracts [92] or alternative routes mediated by Rho protein-independent EC destabilization. Proteins similar to Rho have been found in mycobacteria, suggesting that termination mechanisms dependent on Rho or similar proteins, such as Mfd, are still unknown in mycobacteria [93].

Transcription terminators are key for designing replicative or integrative vectors in rBCG. Although not entirely necessary, the presence of a stem-loop (rich in G-C) and an imperfect U-trac downstream of the stop codon is recommended for inclusion in constructs intended for the expression of mycobacterial proteins. When different *E. coli* canonical sequences were included for in vitro expression of both *E. coli* and *Mtb* proteins [92], transcription termination in mycobacteria was not possible using terminators lacking U-tracts. In agreement, despite the presence of the termination factors NusA and NusG, imperfect U-tracts are found in the *Mtb* genome, making the role of U-tracts ambiguous in mycobacteria. In terms of terminator sequences, the construction of the expression vector should include termination sequences with imperfect U-tracts between position +1 and +50 downstream of the termination codon [91].

### 2.4. BCG Codon Optimization

Optimal expression of heterologous proteins implies an adequate balance between the strength and mechanism of promoter action, efficient transcription termination, and adequate reading of the sequences by ribosomes during the gene translation. Therefore, taking into account the high G+C content in the mycobacterial genome (~65%), codon optimization is pivotal for the optimal expression of non-mycobacterial proteins in rBCG. Strategies aimed at optimizing the immunogen DNA-coding sequence have proven efficacy in shaping the elicited immune response. The use of mycobacterial optimal codons can increase the transcriptional/translational activity of the foreign gene and correlate with enhanced immunogenicity of rBCG expressing codon-optimized p24 compared to native p24 [94]. Another study demonstrated that only when the rBCG-gag was codon-optimized as a priming candidate, in combination with a recombinant vaccinia virus boost containing the native gene, did it produce significantly enhanced IF-ɣ and IL-2 secretion. These responses seemed to be dose-responsive to the codon-optimized immunogen [95]. As well as BCG, *Mtb* is useful for the heterologous expression of proteins for vaccine purposes. Recently, the attenuated TB vaccine MTBVAC was used as a recombinant vehicle to deliver antigens (toxins) of diphtheria, tetanus, and pertussis (the immunogenic constituents of the DTP vaccine), using a combined strategy of integrative promoters based on the mycobacteriophage L5 (ensuring plasmid stability), the inclusion of FLAG epitopes in frame with the C-terminus to facilitate antigens identification, and the optimization of signal sequences and codon usage [96]. In this case, the codon optimization was based on replacing T/A with C in the wobble position for the amino acids Tyr, Ser, Ile, Ser, Asn, Phe, Val, and Ser, as well as A with G in Lys [96].

It was also possible to elicit MHC class I and II immune response in mice through immunization with recombinant vaccinia virus/rBCG expressing the in silico-optimized codon HIV1 CRF01_AE *gag* gene for *Mycobacterium*. Thus, the codon-optimized protein was expressed 11-fold compared to the expression observed by the native HIV-1 Gag protein in the same expression system. When performing codon optimization, prime-boost immunization produces significantly enhanced IFN-γ and IL-2 secretion, which means that optimization benefits a response mediated by CD4+ and CD8+ T cells [95]. Similar results were also obtained by codon modification of the HIV type 1 p24-Gag protein expressed in rBCG, which showed 40-fold more expression levels than non-optimized native protein [94]. Specifically, the G+C percentage of the native gene (43.4%) increased to 67.4% by changing A/T to C in the third position of the amino acids Arg and Leu. This codon modification was effective in eliciting antigen-specific CD8+ T-cell response. By contrast, despite increasing protein expression of the *Schistosoma mansoni* Sm14 antigen 4-fold through codon optimization in rBCG, immunization with the recombinant strain produced cell-specific TH1-predominant immune response (IFN-γ production) in splenocytes only to similar levels of animals immunized with rBCG expressing the native gene [97].

### 2.5. Antibiotic-Free Selection Systems

Antibiotics are commonly used for selecting recombinant clones and protein expression in vitro. However, the regulatory requirements of health authorities limit the use of antibiotics in recombinant vaccines or live vectors for therapeutic agents [39,98]. The absence of antibiotic-resistance genes prevents the spread of antibiotics in the environment and the transfer of resistance to pathogenic strains [98,99]. For recombinant live vaccines such as rBCG, selection pressure is lost in vivo, preventing vaccine stability and the induction of long-lasting immune responses [39,53]. Therefore, alternative selection methods have been developed to overcome the problems associated with antibiotic selection. Alternative selection systems include complementation of auxotrophic markers, post-segregational killing, RNA interference, and chromosomal integration [98,99].

Systems based on auxotrophic complementation have been widely used to construct rBCG strains and are useful for the development of recombinant live vaccines [39,98]. Narcis et al. (2012) [100] constructed an *E. coli*-*Mycobacterium* shuttle plasmid pJH222.HIVA^CAT^ expressing the HIV-1 clade A immunogen HIVA. This shuttle vector employs an antibiotic resistance-free mechanism based on the Operator-Repressor Titration (ORT) system for plasmid selection and maintenance in *E. coli* and lysine complementation in mycobacteria [100]. This shuttle plasmid was electroporated into the parental lysine auxotrophic (safer) strain of BCG to generate the BCG.HIVA^CAT^ vaccine. The BCG.HIVA^CAT^ vaccine, in combination with MVA.HIVA, induced HIV-1 and *Mtb*-specific INF-γ producing T-cell responses in newborn and adult BALB/c mice [100].

On the other hand, Narcis Saubi et al. (2014) [101] developed a novel mycobacterial vaccine design by using an antibiotic-free plasmid selection system. They constructed an *E. coli*/*Mycobacterium* shuttle plasmid p2auxo. HIVA, expressing the HIVA immunogen [101]. This vector employs an antibiotic resistance-free system for plasmid selection and maintenance, which relies on amino acid complementation: glycine in *E. coli* and lysine in mycobacteria. The episomal replicative p2auxo.HIVA vector showed stability in vivo after 7 weeks of mouse immunization [101]. The BCG.HIVA^2auxo^ vaccine strain prime, combined with the MVA.HIVA boost, was safe and induced specific HIV-1 and *Mtb* immune responses in BALB/c mice. Therefore, the *E. coli*/*Mycobacterium* shuttle vectors, which are based on double auxotrophic complementation and an antibiotic-free selection system, provide improved tools for mycobacterial vaccine design and bacterial live recombinant vaccine vehicles [101].

Hart et al. (2015) [102] employed the BCG∆*leu*CD strain to express the HIVgp120 and simian immunodeficiency virus (SIV) Gag proteins, using selectable leucine auxotrophic complementation [102]. The authors demonstrated vaccine stability, ensuring successful antigen expression in subsequent passages, both in vitro and after 60 days of animal inoculation. This was correlated with the induction of a long-lasting immune response in a mouse model [39,53,101]. Moreover, recombinant pantothenate auxotrophic strains of BCG (BCG∆*panCD*) expressing Gag, HIV-1 subtype C reverse transcriptase (RT), or truncated Env (Gp120) HIV-1 proteins were used to prime vaccinate mice, followed by boosting with MVA expressing the same antigens. All rBCG strains were stable in vivo and in vitro, and induced HIV-1-specific T-cell responses [39,103]. Thus, vaccine vectors derived from auxotrophic strains may provide safety and stability, enhancing the induction of long-lasting immune responses.

Another strategy used in antibiotic-free systems is the use of thermosensitive counter-selectable vectors. For example, the thermosensitive (Ts) version of the pAL500 replicon is replicative at all temperatures in *E. coli*, but only at a permissive temperature of 30 °C in *M. smegmatis* [104]. Ts plasmids have been used for site-specific integration and allelic exchange in *M. smegmatis* but are not adequate in *Mtb* due to the limited temperature range of this bacterium [105]. Therefore, a two-step strategy was developed using the Ts vector in combination with the *sacB* gene, which encodes the enzyme levansucrase, which is toxic to bacteria in a medium containing sucrose. *SacB* is used as a counter-selective marker to select for both single-crossover and illegitimate recombination events [105,106,107].

### 2.6. In Vitro and In Vivo Genetic Plasmid Stability

Avoiding strong promoters or using integrative vectors are well-known strategies to prevent genetic instability during homologous or heterologous expression of proteins in rBCG [43]. In the same way, the presence of antibiotic-resistance genes as selection markers contributes to genetic instability during gene expression and inefficient long-term immune response [108] and is restricted in live vaccines to be tested in clinical trials.

As mentioned above, the complementation of a *Mtb* mutant defective in the Ca^2+^ transporter CtpF (*Mtb*∆*ctpF*) was not possible in an MH-S macrophages model of infection using the replicative vector pMV261 [76]. Although heat treatment (45 °C for 1 h) or reactive oxygen species (ROS) production by infected macrophages are sufficient conditions to induce the *hsp60* promoter, the parental phenotype was not recovered in the complemented strain (*Mtb*∆*ctpF*:*ctpF*). Specifically, the *ctpF* gene expression levels increased 58-fold in the complemented *Mtb*Δ*ctpF*:*ctpF* strain compared to the wild-type strain (*Mtb*) at 7 days after infection, indicating that overexpression of *ctpF* was detrimental to the mycobacterium [76], as also observed in other studies [53,54,103].

Therefore, the use of integrative vectors derived from mycobacteriophages such as L5 is a good option to reduce genetic instability by reducing the metabolic burden [44]. Genetic instability, which also occurs in L5-based integrating vectors, can be prevented by including an *attP* site in *cis* and an integration function in *trans.* This increases the stability of the integrative vectors in the *attB* site, and considerably reduces the excision of sequences inserted into the mycobacterial genome [109].

A comparative study evaluated the heterologous HIV-1gp120 gene expression in BCG employing pMV261 (regulated by *hsp60* promoter), pJH222, and pJH223 (both controlled by the α-antigen *Mtb* promoter) vectors [59]. Meanwhile, pMV261::HIVgp120 displayed disruption of the HIV-1gp120 gen; by contrast, pJH222::HIVgp120 and pJH223::HIVgp120 did not show any genetic disruption. In this case, it has been proposed that genetic instability could be linked to the presence of potential glycosylation sites within the protein encoded by the target gene, as was observed for the expression of the HIV-1gp120 protein using replicative vectors in rBCG [59,105].

Mahant et al. (2017) [72] constructed the integrative plasmid p2auxo.HIVA^int^ expressing HIVA immunogen in the BCGΔ*lys* strain to generate the vaccine BCG.HIVA^2auxo.int^ [72]. This expression vector uses an antibiotic-free selection system. They demonstrated that the in vitro stability of the integrative plasmid p2auxo.HIVA^int^ was enhanced 4-fold as compared to the BCG strain containing the episomal plasmid. The BCG.HIVA^2auxo.int^ vaccine, in combination with MVA, was safe and induced HIV-1 and *Mtb*-specific INF-γ T-cell responses in adult BALC/c mice [72]. Similar behavior was observed by Kilpeläinen et al. (2019) [110] when they characterized the rBCG expressing the “HIVACAT T-cell immunogen” (HTI), a mosaic immunogen designed by grouping HIV-1 T-cell epitopes identified in patients with low HIV-1 viral loads [110] in integrative vector p2auxo^int^ [10]. The BCG.HTI^2auxo.int^ strain was shown to be stable in vitro for 35 bacterial generations, and, when delivered in combination with Oxford chimpanzee adenovirus (ChAdOx1).HTI, it induced and increased the HIV-1-specific T-cell responses in adult BALB/c mice [10]. Therefore, employing integrative expression vectors along with an antibiotic-free plasmid selection system that relies on “double” auxotrophic complementation is expected to enhance the stability and immunogenicity of mycobacterial vaccines in vivo [72].

Méderlé et al. (2002) [55] constructed rBCG strains co-expressing the early regulatory *nef* and the structural *gag* (p26) genes from SIV, separately into a pAL5000-derived replicative vector and an Ms6-derived integrative vector, and the expression levels were evaluated in a mouse model [55]. In these cases, the gene expression via integration into specific sites of BCG chromosome showed higher genetic stability after 100 days of inoculation in mice (98% of colonies recovered from spleens had retained both *nef* and *gag* expression), compared with only 25% when the replicative vector was used [55]. In addition, the anti-Gag INF-γ producing CD4+ T cells were only detected 6 weeks after inoculation in mice vaccinated with the integrative rBCG strain, suggesting the importance of sustained and stable antigen expression to achieve a long-lasting immune response [53,55].

The antibiotic-free selection system based on auxotrophic complementation also improves in vivo stability [53,72]. Borsuk et al. (2007) reported the construction of an rBCG using an auxotrophic complementation system without antibiotic-resistance markers, showing high stability even during in vivo growth due to the persistence of selective pressure, whereas the conventional vectors were unstable in the absence of selective pressure [72,111]. Another study showed that the episomal replicative pJH222.HIVA^CAT^ unmarked antibiotic vector based on the Operator-Repressor Titration (OTR) system was stable in vivo over 20 weeks after mouse immunization and in combination with MVA.HIVA.

In conclusion, to prevent genetic plasmid instability both in vivo and in vitro, different approaches should be considered: (i) the use of an expression vector containing small foreign DNA sequences; (ii) DNA fragments lacking glycosylation sites; (iii) the use of weak or inducible promoters; (iv) the use of BCG auxotrophic strains containing complementation genes; (v) mycobacterial codon optimization of the recombinant gene; (vi) the choice of the expression vector; and (vii) the prevention of foreign protein toxicity to the bacteria [59,72].

### 2.7. Targeting of Heterologous Antigens

Traditionally, BCG has been used for heterologous expression of mycobacterial antigens as a vaccine strategy; however, its intrinsic capacity to stimulate a broad CD4+ and CD8+ T-cell and humoral response has led to its use as a vaccine vehicle to induce immune responses to other bacterial antigens, parasites, or viruses. Although low doses of BCG bias Th1-type immune responses, which are important for controlling intracellular infections, several attempts have been made to express intracellular/extracellular parasite antigens. For example, the *Plasmodium falciparum* circumsporozoite protein (CSp) expressed in rBCG has shown upregulation of MHC class II activation and IFN-γ producing memory cells and antibodies in mice [112].

Moreover, rBCG expressing the Sm14 antigen of *Schistosoma manosi* induces predominantly Th1-type response [113]. In contrast, BCG expressing the *Toxoplasma gondii* cyclophilin (TgCyP) induces both Th1 and Th2 responses in BALB/c mice [114], a finding that is analogous to the responses observed in BCG expressing proteins of *Trypanosoma cruzi* [115] and *Efímera maxima* [116]. Similarly, rBCG antibacterial vaccines elicit adequate cellular and humoral responses. This phenomenon is found by the expression of Leptospira antigens, which elicit a cellular immune response [117], while the expression of *Borrelia burgdorferi* results in the generation of high antibody titers in mice [22]. Likewise, rBCG expressing Bordetella pertussis antigens induced cellular and protective IgG and mucosal IgA responses in mice [118,119].

Concerning the heterologous expression of viral antigens in rBCG, research in the field of HIV vaccine development has indicated that broadly neutralizing antibodies, cellular responses, and mucosal and innate immunity play a pivotal role in the protection against HIV. Initially, rBCG expressing the DNA-coding sequences corresponding to HIV *gag* or *env* genes induced strong neutralizing antibody responses. Furthermore, the immune responses elicited by rBCG significantly reduced the viral load in macaques challenged with a non-pathogenic simian-human immunodeficiency virus (SHIV), compared to non-vaccinated control animals [120,121]. In several studies, conserved epitopes or consensus sequences of HIV-1 proteins, including Env gp120, Env gp41, Tat, and Nef, have been selected for expression by rBCG [34]. However, in the context of HIV vaccine development, it is crucial to consider the antigenic variability of viral proteins, which presents a significant challenge in targeting epitopes. In the case of HIV, these variations can be attributed either to point mutations during reverse transcription, influenced by immune selection, or to recombination events. Consequently, eliciting an efficacious immune response to HIV antigens has proven challenging, and they have not been sufficient to confer efficient protection [34,120].

Lim et al. (1997) [122] showed that the rBCG strain expressing a large N-terminal portion of the SIVmac251 Env gp110-encoding gene induced strong specific cytotoxic T lymphocytes in mice and humoral immune responses in mice and guinea pigs. Additionally, the anti-gp110 IgGs produced were able to neutralize in vitro growth of virulent SIVmac 25a isolates [122]. Alternatively, a promising approach to induce broadly neutralizing antibodies (bnAbs) is the use of stabilized soluble native-like HIV-1 Env trimers, such as the SOSIP.664 gp140 trimer [34,123]. Additionally, the identification of multiple broadly neutralizing antibodies (bNAbs) targeting the HIV-1 envelope glycoprotein (Env) trimer has enabled its structural characterization, the Env immunogen design, and the development of an HIV-1 vaccine candidate [123]. It has been demonstrated that native-like Env trimer can induce autologous Nab responses against resistant (Tier-2) viruses in several animal models [123].

Furthermore, the immunogens tHIVconsvX have been used for the design of vaccines due to their capacity to combine the principal strategies for elicitation of effective CD8+ T cells: (i) the use of regions of HIV-1 proteins functionally conserved; (ii) the incorporation of mosaic immunogens to maximize global epitopes; and (iii) the inclusion of epitopes that are associated with low viral load in infected untreated individuals. The CD8+ T-cell responses to tHIVconsvX-derived peptides in treatment-naïve HIV-1+ patients were found to be correlated with a high CD4+ T-cell count and low viral load [124]. Accordingly, an interesting strategy for HIV-1 vaccine development is the use of mosaic and conserved immunogen regions to enhance the coverage of global HIV-1 variants and induce both bNAbs and effective CD8+ T-cell responses.

### 2.8. Antigen Localization

Heterologous antigen localization is an important issue to consider for recombinant BCG-based HIV vaccine development. The accumulation of heterologous proteins in the cytoplasm of mycobacteria may produce deleterious effects. There are several strategies for antigen localization when expressing proteins in BCG. In this sense, the secretion of the expressed proteins, their localization on the bacterial surface, and their intracellular production determine the mechanism of antigen presentation and the type of immune response that will be elicited against the heterologous antigen [125]. Some antigens may function optimally when expressed within the cytoplasm of BCG. This is particularly relevant for antigens that require processing and presentation via the MHC pathway. Although intracellular expression can produce cellular cytotoxicity and delayed T-cell stimulation, this led to antigen presentation to CD4+ and CD8+ T cells eliciting cellular immune responses. For example, in the VPM1002 BCG vaccine where the urease gene was replaced with a listeriolysin from *L. monogytogenes* (BCGΔureC::hly) (urease depletion allows phagosome acidification and phagolysosome fusion), the intracellular expression of listeriolysin O in VPM2 promoted bacterial lysis, autophagy, inflammasome activation, and apoptosis [126].

Alternatively, the expressed protein can be anchored to the BCG cell wall or plasma membrane, promoting direct interaction of the protein with host immune cells. Therefore, the fusion of the heterologous protein to surface-exposed proteins or cell-wall components of BCG is convenient for enhancing antigen presentation and immune recognition. For example, the foreign protein can be anchored to cell membrane by fusion with the signal sequence of the 19 KDa lipoprotein of *Mtb* [127]. Indeed, the M protein of porcine reproductive and respiratory syndrome virus (PRRSV) fused to the signal sequence of the *Mtb* 19 KDa protein directed the expression of the fused protein to the BCG cell surface, generating a recombinant strain that was able to produce neutralizing antibodies against the porcine virus [128].

Furthermore, several studies have used leader peptides to facilitate protein secretion into the extracellular environment. Thus, proteins enhance their accessibility to host immune cells, improving the immune responses. In a pioneer study, a large N-terminal fragment of the SIVmac251 Env gp110 protein was fused to the signal sequence of the *β*-lactamase *M. fortuitum* protein. The fusion protein was secreted after cleavage of the signal sequence, and this rBCG strain was able to induce a strong CTL response in mice and a humoral response in mice and guinea pigs [122]. Similarly, the signal sequence of Ag85 [58,129] and the mycobacterial exported repeated protein (ERP) have been used to express secreted proteins in rBCG [129]. Mice immunized with an rBCG strain expressing the MSP1 antigens of *Plasmodium yoelii* fused to the Ag85 signal sequence of *M. kansassi* induced higher protection against blood-stage parasite infection than the recombinant MSP1-15 protein combined with Freund’s adjuvant [20].

## 3. Specific SIV/HIV Immune Responses in Small Animal Models and Non-Human Primate Models

The evaluation of the immunogenicity in small animal models, such as mice and guinea pigs, and further studies in non-human primates are crucial to compile information on how the vaccine may perform in humans and for vaccine strategy optimization. In this section, we outline in vivo immunogenicity studies of rBCG expressing SIV and HIV antigens, focusing on those carried out since 2011. Previous animal trials have been reviewed extensively [46,67]. A summary of animal trials conducted after 2011 is shown in Table 1, considering the following characteristics: (1) the level of antigen expression; (2) the type of expression vector (replicative vs. integrative, promoter, signal sequences); (3) genetic plasmid stability; (4) the immunogen design; (5) the mycobacterial strain; and (6) the dose and route of immunization. In mouse models, BALB/c mice are the most commonly used hosts, followed by C57BL/6 mice.

### 3.1. Small Animal Models

The ideal rBCG-based HIV vaccine should generate robust and multifunctional T-cell responses, durable mucosal memory T cells, and neutralizing antibodies. rBCG, as a vector, has been demonstrated to elicit HIV-1 specific neutralizing antibodies and T-cell responses in small animal models [34,130,131,132]. Moreover, BCG’s potential as an inducer of mucosal immunity is crucial since the primary route of HIV entry is through the mucosal surface. rBCG-gp120 has also been shown to induce mucosal immunity against HIV antigens in the vaginal and respiratory tracts in murine models [133].

Since attenuated live bacterial vaccines often lacking immunogenic potential on their own, several types of priming-boosting vaccinations have been assessed. Several combinations of rBCG expressing HIV antigens coupled with viral boosting vectors have been shown to induce HIV-specific CD8+ and CD4 + T-cell responses [61,102,103,134]. Incorporating viruses as a booster agent, such as modified vaccinia virus Ankara-vectored (MVA) or Ovine Atadenovirus-vectored (OAdV), has elicited robust specific HIV T-cell responses [135]. Heterologous rBCG-Gag/MVA-Gag regimen increase the secretion of Gag-specific IFN-ɣ in ELISPOT assays compared to the homologous vaccination regimen with either rBCG-Gag or MVA-Gag alone [136]. Although BCG offers many advantages as a live vaccine vehicle for HIV, expressing viral antigens in BCG is often challenging, because of genetic instability and low immunogenicity. Some studies have aimed to generate stable recombinant BCG strains expressing HIV antigens by modifying the HIV gene immunogen. Recombinant mycobacteria expressing HIV-1 Gag fused to GFP were selected to express a modified, more stable Gag antigen in BCG, using directed evolution and selecting for increased growth rates and higher levels of GFP expression. The recombinant BCG that expressed the modified HIV-1 Gag induced 2- to 3-fold higher levels of Gag-specific CD4 T cells than those expressing the unmodified Gag and MVA-Gag boost conferred protection to a surrogate vaccinia virus challenge [88]. Moreover, the addition of a CD1d-restricted natural killer T (NKT) cell-activating glycolipid to rBCG strains expressing an SIV Gag antigen (rBCG-SIV gag) has been shown to boost gag-specific CD8+ T-cell responses in C57BL/6 mice and partially humanized mice expressing human CD1d molecules [137].

Antigen compartmentalization is another factor that can significantly influence the induction of the immune response. The high concentration of heterologous proteins in the cytoplasm of mycobacteria may produce a deleterious effect and high metabolic burden. Many studies have described the use of leader peptides to target the protein to the mycobacterial membrane or facilitate the protein secretion, and this has been associated with increased stability, enhanced expression, and higher immunogenicity [62,108]. It has also been suggested that removal of the foreign antigen from the mycobacterial cell by secretion may support higher levels of its production [138]. Other research groups using rBCG have reported improved immune responses and protection by binding the foreign antigen to the 19 kDa signal sequence [19,121,139,140].

Modification of BCG to improve its safety is another important aspect, as assessed by Chapman R. et al. (2012) [108]. The attenuated pantothenate auxotrophic BCG strain (BCG-pan) was evaluated against wild-type BCG Pasteur for their effectiveness as vaccine vectors for HIV-1 subtype C Gag in BALB/c mice. The BCGpan-Gag prime induced high frequencies of Gag-specific CD8 + T cells as compared to the BCG-Gag prime, which mainly induced Gag-specific CD4 + T cells. Vaccinated mice were protected against surrogate viral challenge consisting of vaccinia virus expressing HIV-1 DU422 Gag (Clade C), and a safer profile and reduced inflammation was observed, suggesting the advantage of using auxotrophic BCG strains, such as the BCGpan-Gag, over parental strains [108].

The influence of age, dose, and immunization route are also critical factors that play an important role in the outcome of the immune responses. Our group has evaluated the influence of age and immunization routes for the induction of HIV-1-specific immune responses after neonatal (7 days old) and adult (7 weeks old) BALB/c mouse immunization with BCG.HIVA(222) prime and MVA.HIVA boost. The frequencies of HIV-specific CD8(+) T cells producing IFN-γ were higher in adult mice vaccinated intradermally, while they were lower in both adult and newborn mice vaccinated subcutaneously. On the other hand, when the HIV-specific CTL activity was assessed, the frequencies of specific killing were higher in newborn mice than in adults [63]. Promkhatkaew et al. (2009) have explored the potential of recombinant Vaccinia virus DIs strain harboring the same HIV-1 CRF01_AE gag gene (rVaccinia/ HIV-1gagE) present in the BCG construct, using different immunization routes [95]. Elevated CTL responses were observed one month after a single subcutaneous (s.c.) injection of rBCG/HIV-1gagE, targeting various epitopes across the entire Gag protein, compared to the intradermal (i.d.) route. A prime-boost regimen having only rDIs/HIV-1gagE injected i.d. induced very low CTL levels. However, within two months, by priming with rBCG/HIV-1gagE s.c. and boosting with rVaccinia/HIV-1gagE intravenously (i.v.), CTL levels were greater than those obtained by priming and boosting both i.d. [95]. Although the route of immunization plays an important role in the outcome of the immune responses, other factors, such as level of heterologous protein expression and dose of BCG delivered, should be considered. Although most studies carried out using small animal models utilize a dose of 10^6^–10^8^ CFU (Table 1), lower doses have also been assessed [100,136]. A BCG-pan strain expressing the HIV-1C mosaic Gag (GagM) immunogen delivered at a dose of as low as 10^4^ CFU combined with MVA was assessed pre-clinically in a prime-boost regimen in BALB/c mice and was shown to boost responses against the mosaic HIV-derived immunogen, resulting in strong specific cellular immune responses [136].

Apart from vaccine immunogenicity, the safety profile of rBCG:HIV strains is also a critical issue to consider. Numerous studies have described the safety profile in adult and newborn mice, with extremely positive outcomes. For safety profile assessment, body mass was monitored over time and recorded to depict any adverse events and body mass loss due to vaccination [63,65,101,141].

### 3.2. Guinea Pigs

A rBCG expressing full-length SIV-Gag was tested in guinea pigs via both i.d. and oral routes. T-cell proliferative responses to both SIV Gag p27 and PPD antigens were observed in animals immunized either i.d. or orally. However, the PPD-specific responses were notably higher in guinea pigs immunized via i.d. compared to those immunized orally. An augmentation in the expression of IFNγ mRNA specific to both PPD and SIV Gag p27 was also observed in both immunization groups that received rBCG-SIVGag. In addition, both i.d. and oral immunization with rBCG-SIVGag induced PPD- and SIV Gag p27-specific serum IgG responses [142]. Following this study, Mamorou Kawara et al. (2008) [143] assessed the durability of the induced immune responses over a three-year period. Gag-specific serum IgG was consistently generated at high levels for 3 years in guinea pigs (IgG2 > IgG1) after i.d. and oral immunization. The increase in IFN-γ mRNA expression following in vitro restimulation with the Gag antigen was also observed in PBMC from both immunization groups over the entire 3-year observation period. In guinea pigs immunized i.d. with rBCG-SIVgag, a high Gag-specific IFN-γ response was detected one year after vaccination, though this response gradually declined over time [143].

### 3.3. Non-Human Primates

A large number of in vivo studies of mycobacterium expressing SIV/HIV-1 immunogens have been performed in non-human primates to evaluate the immunogenicity and protection after viral challenge (Table 1). Non-human primates have close phylogenetic proximity to humans and their immune system is comparable to that of humans, with high sequence homology between components of the human immune system and that of non-human primates [144,145,146,147,148,149,150,151,152].

Immunization with rBCG expressing SIVmac Gag protein under the control of *hsp70* regulatory sequences elicited MHC class I-restricted, CD8+ SIVmac gag-specific CTL in rhesus monkeys [153]. Yasutomi et al. (1995) demonstrated that a potent SIV-specific CTL response can be elicited by combining live vector and peptide vaccine modalities [154]. However, a CTL response specific to a single SIV Gag epitope (mapped to residues 182–190 of the Gag protein, p11C), in the absence of SIV-specific antibodies, failed to confer protection against a cell-free, intravenous SIV challenge.

For the first time, rBCG constructs harboring the four major open reading frames of the SIV genome (*gag*, *pol*, *env*, and *nef*) were combined into a single inoculum and administered to rhesus macaques intravenously (iv). The results demonstrate the induction of SIV-specific systemic IgA and IgG antibody and cellular immune responses, including CTLs and helper T-cell proliferation [155]. A single i.d. administration of three recombinant BCG strains expressing the SIVmac251 *nef*, *gag* and *env* genes in cynomolgus macaques induced CTL responses and IFN-ɣ secretion. A rectal or oral boosting dose of rBCG-SIV elicited anti-SIV IgAs in the rectum of vaccinated monkeys, but not IgG antibody responses or lymphoproliferation against the SIV antigens in blood [156]. Immunization with rBCG expressing an HIV-1 Env V3 antigen (rBCG Env V3) elicited significant levels of V3 NAb in rhesus macaques. Although this response did not confer protection against a pathogenic SHIV challenge, it significantly reduced the viral load in macaques after exposure to a non-pathogenic SHIV [121].

An enhancement of the immunogenicity of HIV-1 and SIV antigens expressed by rBCG and other mycobacterial vectors has been achieved through a heterologous prime-boost immunization strategy. When cynomologus macaques were primed with rBCG-SIVgag and then boosted with rDIsSIVgag, high levels of IFN-ɣ spot-forming cells specific to SIV Gag were induced. This combination regime elicited effective protective immunity against mucosal challenge with the pathogenic SHIV virus for 1 year during which the macaques were under observation [157]. Cayabyab et al. (2009) also explored whether they can augment the immune responses elicited by rBCG expressing SIV Gag, Pol, and Encv antigens by boosting with a replication-defective rAd5 vector [158]. They demonstrated that i.d. and iv administration of rBCG are better tolerated than other routes of rBCG administration, and that rBCG prime/rAd5 vector boost vaccination induced robust, persistent, and polyfunctional CD8 T-cell responses [158]. In line with these prime-boost studies, Chege et al. (2009) [159] studied the immune response in baboons following an initial vaccination with rBCG constructs that expressed the *gag* gene from a South African HIV-1 subtype C isolate, followed by a booster with HIV-1 subtype C Pr55(gag) virus-like particles (Gag VLPs). They demonstrated that while these rBCG constructs alone elicited only weak or undetectable HIV-1 Gag-specific T-cell responses, they effectively primed for a subsequent boost with Gag VLPs, which strengthened and broadened the immune responses. In addition, a Gag-specific humoral response was elicited [159]. On the other hand, a novel vaccine designated BCG.HIVA401, vectored by AERAS-401, expressing the HIVA immunogen, demonstrated priming of T cells to the HIVA transgene product in rhesus macaques and was readily boosted with MVA.HIVA and OAdV.HIVA vaccines to elicit broad and robust HIV-1-specific T-cell responses [135]. This rBCG was also tested in infant rhesus macaques using a prime-boost regimen that included BCG.HIVA401, followed by recombinant modified vaccinia virus Ankara (MVA.HIVA). The regimen was shown to be safe, although it elicited lower T-cell immunogenicity compared to that observed in adult animals. This was the first time these vaccines had been tested in newborn monkeys [160]. In infant macaques, recombinant attenuated *Mtb* strain mc26435 expressing SIV Gag and harboring attenuations of genes critical for replication (*panCD* and *leuCD*) and immune evasion (*secA2*) priming, in combination with MVA-SIV boosting, induced low levels of SIV-specific immunity after oral and i.d. priming, which were enhanced following the boosts. SIV-specific plasma IgG and IgA, mucosal IgA, as well as intestinal IgA and IgG antibodies were detected in animals immunized with this regimen [161].

Kato et al. (2021) developed a urease-deficient BCG strain Tokyo172 (BCGΔurease) to enhance its immunogenicity [162]. BCGΔurease expressing a SIV Gag elicited more efficient BCG antigen-specific CD4 and CD8 T-cell responses, as well as a higher number of Gag-specific CD8 T cells. Cynomolgus monkeys were primed with rBCG expressing SIV genes, followed by a boost with SIV gene-expressing LC16m8Δ vaccinia virus and a second boost with SIV Env-expressing Sendai virus. Following repeated intrarectal challenges with a low dose of SIVmac251, two out of six vaccinated animals were protected, while all seven control animals became infected [162].

In order to evaluate if the Chacma baboon model may serve as a valuable tool for evaluating the immunogenicity of antigens expressed by rBCG vaccines, they assessed the immune response of Chacma baboons to both the Tokyo and Pasteur strains of BCG. Although a humoral immune response to BCG was observed in only a subset of vaccinated baboons, a cellular immune response characterized by a PPD-specific delayed hypersensitivity reaction and BCG-specific IFN-ɣ production from PBMC was consistently detected. This response persisted for over a year after a booster inoculation at 20 weeks, suggesting that baboons might serve as a NHP model for evaluating rBCG-based vaccines [163].

rBCG constructs expressing SIV Gag protein were compared to recombinant BCG constructs containing specific heterologous gene deletions identified through an in vitro screening process aimed at enhancing immunogenicity. Their findings demonstrated that BCG-SIVgag is capable of inducing strong transgene-specific priming responses, resulting in strong SIV epitope-specific cellular immune responses. While enhanced immunogenicity was sustained at moderate levels for >1 year following the heterologous boost vaccination, they were unable to demonstrate a protective effect after repeated rectal mucosal challenges with pathogenic SIVmac251 [13].

Immunodominance significantly limits the capacity to produce broadly targeted, HIV-specific cellular immune responses through vaccination. Martins et al. 2014 [14] attempted to circumvent this phenomenon and thereby broaden the repertoire of SIV-specific cellular responses by vaccinating rhesus macaques with minigenes encoding fragments of Gag, Vif, and Nef [14]. Unlike earlier mouse studies, this approach had little impact on CD8 T-cell immunodominance hierarchies in monkeys, as evidenced by the detection of only one subdominant epitope in Mamu-A*01 vaccines. This highlights the challenge of inducing subdominant CD8 T-cell responses through vaccination and suggests that approaches beyond gene fragmentation may be necessary to substantially shift immunodominance in primates. While some of the tested regimens were highly immunogenic, their efficacy was limited to a slight reduction in set-point viremia following the SIVmac239 challenge. No protective correlates were identified, reinforcing the idea that vaccine immunogenicity does not necessarily predict the ability to control AIDS virus replication [14].

**Table 1:** Specific SIV/HIV immune responses induced after rBCG:SIV/HIV immunization using different animal models.

**1.1. Murine and guinea pig model**						
**Animal Model**	**Recombinant Antigen**	**Promotor, Signal Sequence and Expression Vector**	**Route**	**rBCG Dose/Boost**	**Immunity Elicited**	**Reference**
Mice (BALB/c)	HIVA	Ag85B promoter, 19-kDa signal peptide, pJH222	IP	10^6^ CFU rBCG + 10^6^ HAdV5.HIVA/10^7^ OAdV7.HIVA/100 µG pTH.HIVA DNA IM	Both strains elicit specific CD8 T-cell responses; HAdV5.HIVA and OAdV7.HIVA promoted the most robust responses and different intercellular signaling molecules in splenocytes between strains.	[134]
Mice (BALB/c)	HIV-1 V3-concatemer	hsp60 promoter, pMV261	IP	10^7^ CFU rBCG	rBCG promoted anti-mV3 polyclonal antibodies but the antiserum did not block HIV-1 replication. Additionally, vaccination was able to induce and maintain memory T cells.	[164]
Mice (BALB/c)	HIV-1 gag (Subtype C), RT or gp120.	mtrA promoter, 19-kDa signal peptide, pCONEPI	IP	3 × 10^7^ CFU + MVA expressing polyprotein Gag, RT, Tat, Nef 10^4^ PFU.	Vaccination boosted with SSAVI MVA-C greatly increased immune response and elicited robust cellular immune response and secretion of IFN-c, TNF-ɑ, and IL-2 against HIV.	[103]
Mice (C57BL/6)	HIV-1 gp120 SIV Gag	Paph, leuCD, PgroEL2, lpqH or fbpB signal sequence, pSL70/ pSL718/ pSL720/ pSL509	RO IM	10^7^ CFU, 3 × 10^7^ rADV5 VP	Leucine auxotroph rBCG-gp120 ensures full-length expression stability after 1024-fold amplification in vitro and 60 growing days in vivo and promotes robust cellular response mediated by T cells.	[102]
Mice (BALB/c)	HIV-1 Subtype C Mosaic Gag	mtrA, 19 kDa signal sequence, pTJBCG3	IP	2 × 10^7^ CFU + 10^2^/10^4^/10^6^ PFU MVA	MVA-Gag^M^ boost induced strong Gag-specific response and long-lasting CD4^+^ and CD8^+^ T cells expressing effector memory phenotype.	[136]
Mice (BALB/c)	HIV-1 p24 Gag	hsp65 promoter, pMyong2	SC	10^6^ CFU	rBCG elicited a higher antibody production, gag-specific immune response, and more robust CTL response than rSmeg.	[11]
Mice (BALB/c)	HIVA	ɑ-antigen promoter, 19-kDa signal peptide, pJH222	ID	10^6^ CFU + 10^8^ IU ChAdOx1.tHIVconsv5&6	Integrative expression vectors increased vaccine stability in vitro and in vivo, were well tolerated, and elicited T-cell responses.	[110]
Mice (Balb/c + SCID)	HIVA	ɑ-antigen promoter, 19-kDa signal peptide, pJH222	IDIP	10^6^ CFU rBCG + 10^6^ PFU MVA.HIVA 10^6^ CFU	Vaccination with MTBVAC.HIVA2auxo boosted with MVA.HIVA promoted polyfunctional HIV-1-specific CD8^+^ T cells with high production of IFN-γ, TNF-ɑ, and CD107ɑ.	[65]
Mice (BALB/c)	HIVACAT T-cell immunogen (HTI)	ɑ-antigen promoter, 19-kDa signal peptide, pJH222	ID	10^6^ CFU rBCG + 10^6^ PFU MVA.HTI	BCG.HTI2auxo.int boosted with MVA.HTI was safe and elicited specific T-cell response with a wider recognition profile.	[141]
Guinea pig	SIVmac239 gag gene	hsp60, pSO246	ID O	0.1 mg rBCG 80 mg rBCG	rBCG expressing SIVGag elicited long-lasting humoral and cell-mediated immunity for bacterial and viral antigens. Specific humoral responses last up to 3 years.	[143]
**1.2. Non-human primates model**						
**Animal Model**	**Recombinant Antigen**	**Promotor, Signal Sequence and Expression Vector**	**Route**	**rBCG Dose/Boost**	**Immunity Elicited**	**Reference**
Rhesus macaques	SIV gag SIV pol SIV env	ɑ-antigen promoter, 19-kDa signal peptide, pJH222	ID IV IM	10^6^ CFU rBCG up to 10^9^ CFU rBCG + 10^10^ vp rAd5	rBCG induced polyfunctional CD8 T cells and rAd5 boosting elicited SIV-specific cellular response.	[158]
Rhesus macaques	HIVA	Ag85B promoter, 19-kDa signal peptide, pJH222	ID	10^7^ CFU rBCG + 5·10^7^ PFU MVA.HIVA IM + 10^10^ IU OAdV.HIVA IM	MVA.HIVA and OAdV.HIVA boosting elicited strong specific T-cell responses against HIV-1 and immunological memory.	[160]
Rhesus macaques	SIVgag	hsp60 promoter, Ag85B or 19-kDa signal peptide, pSL7/pSL10	IV	3 × 10^8^ CFU rBCG + rNYVAC-SIVgag-pol 10^7^ PFU	Vaccination promoted robust specific cellular immune responses against SIV epitopes. No protective immune response was proven during rectal mucosal challenges.	[13]
Rhesus macaques	SIVmac239gag SIVmac239nef SIVmac239vif	Ag85B promoter, 19-kDa signal peptide, pJH222	ID	2 × 10^5^ CFU rBCG	rBCG vaccination regimen elicited better cellular immune response compared to rAd5 and rYF17D, but all the regimens failed to control peak viremia after continuous challenges.	[14]
Rhesus macaques	SIVmac239gag	hsp60 promoter, Ag85B or 19-kDa signal peptide, pMV261	IV	3 × 10^8^ CFU rBCG + 10^7^ pfu NYVAC SIVmac142 gag-pol	No significant differences were found regarding immune response to HIV epitopes depending on preexisting anti-mycobacterial immunity.	[12]
Cynomolgus macaques	SIVmac239 gag	hsp60, pSO246	ID IV	10 mg boosted with 10^6^ PFU rDIsSIVgag	Vaccination with rBCG-SIVgag with rDIsSIVgag as a booster induced strong specific anti-SIV Gag response and protective immunity.	[157]
Cynomolgus macaques	SIVmac239gag SIVmac239gP120 SIVmac239RTN	hsp60 promoter, PSO246	SC	2.5 mg rBCG + m8Δ-SIV-Gag/m8Δ-SIV-gp160/m8Δ-SIV-RT/m8Δ-SIV-RTN 10^7^ PFU.	Challenge with SIVmac251 IR after vaccination and boosting induced protection in two of six animals. The cynomolgus macaques that had the most robust cellular response showed undetectable viremia and in vitro suppressiona activity.	[162]
Chacma baboons	HIV-1 gag (Subtype C)	mtrA promoter or katG promoter, 19-kDa signal peptide, Episomal vector	ID	10^8^ CFU rBCG + 11 µg Gag VLP IM	rBCG elicited robust cellular immune response against HIV-1 epitopes and promoted Gag-specific humoral response.	[159]
Chacma baboons	HIV-1 gag (Subtype C)	mtrA promoter, 19-kDa signal peptide, pHS300/ pHS400/ pRT106	ID	10^8^ CFU rBCG + 10 µg Gag VLP IM	rBCGpan-Gag boosted with Gag VLP elicited strong polyfunctional T-cell response and promoted Gag-specific memory T cells.	[165]

Abbreviations: BCG, Bacillus Calmette–Guérin; rBCG, recombinant Bacillus Calmette–Guérin; HIVA, HIV-1 clade A-derived immunogen; MVA, Modified Vaccinia Virus Ankara; OAdV, Ovine atadenovirus-vectored; ChAdOx, Chimpanzee Adenovirus Oxford; IP, Intraperitoneal; ID, Intradermal; SC, Subcutaneous; RO, Retro-orbital, IM; Intramuscular; O, Oral; IU, International units; CFU, Colony-forming units; PFU, Plaque-forming units; VP, Viral particles; CTL, cytotoxic T-lymphocyte, SIV, Simian immunodeficiency virus; HIV, Human immunodeficiency virus.

## 4. Effect of Preexisting Immunity Against BCG and Viral Vectors on the Responses to Foreign Antigens Expressed in rBCG: Anti-Vector Immunity

An effective immune response to a foreign antigen delivered by a vaccine vehicle could be hindered by an immune response against the vaccine’s vector component. Preexisting immunity to viral vaccine vectors such as adenovirus was shown to have detrimental effects in relation to vaccine efficacy [166,167]. Hence, complementary strategies have been developed. Novel approaches involve the utilization of alternative viral vectors with lower prevalence immunity in the human population, such as the simian adenovirus of chimpanzee origin, ChAdOx1 [110]. Additionally, efforts have focused on identifying adenovirus serotypes that avoid anti-Ad5 immune response. Immunological studies exploring cross-reactivity have pointed to certain serotypes, such as Ad35, which avoids immune cross-reactivity, therefore offering a promising alternative [168]. Finally, modifications of the HAd5 capsid to circumvent recognition by existing antibodies have been described [169]. Meredith L Leong et al. (2009) have shown that, in mice with preexisting L. monocytogenes-specific immunity, priming of naïve T cells was not prevented, and antigen-specific responses could be boosted by additional vaccinations [170].

Because the BCG vaccine has been administered to over two billion people for TB immunization, the effects of previous BCG immunization on subsequent immune responses after recombinant BCG immunization should be considered. One of the first studies was carried out by Marina Gheorghiu et al. (1994) [171]. They analyzed the influence of BCG priming on the growth of rBCG in mice and on cellular and humoral responses induced either against β-galactosidase or against HIV-1 Nef antigen expressed by rBCG [171]. They showed that BCG priming restricts the growth of rBCG in target organs and slightly reduces the proliferative response to foreign antigens. However, BCG-primed mice developed high-level antibody responses against β -galactosidase expressed by rBCG (lacZ) compared to mice never exposed to BCG. These results therefore suggested that BCG immunity in vaccinated individuals will not be a limitation in the use of BCG as a vaccine vector and could even enhance the immunogenicity of future rBCG vaccines [171].

Rosamund Chapman et al. (2010) [67] found that when baboons are given several vaccinations with rBCG expressing Gag, there is an increase in the response to Gag after the second vaccination, suggesting that immunity to BCG does not inhibit responses to the recombinant protein [67]. Similar CTL responses were elicited in BALB/c mice preimmunized and nonimmunized with BCG before administration of rBCG expressing the HIV-1 Env protein V3 region (15 amino acids from Arg315 to Lys329) [172]. Cayabyab et al. (2006) have shown that immunization of mice with recombinant *M. smegmatis* led to the expansion of HIV-1 envelope –specificCD8+ T-cell responses and, importantly, preexisting immunity to *M. bovis* BCG only had a marginal effect on the immunogenicity of recombinant *M. smegmatis* [173].

Rhesus Macaques primed with rBCG constructs expressing SIVgag and boosted with NYVAC-SIVgag-pol [13] were used to assess the effects of preexisting antimycobacterial immunity in the context of rBCG vector immunogenicity [13]. Baseline cellular and humoral mycobacterial immune responses were compared to those of humans from regions with low and high *Mtb* infection prevalence to determine whether prior mycobacterial immunity could influence immune responses. The rBCG-NYVAC prime-boost regimen elicited robust SIV Gag-specific responses, but no correlations between preexisting mycobacterial immune responses and the SIV Gag T-cell responses after vaccination were found [12]. All in all, preimmunization with BCG does not appear to significantly impact responses to foreign antigens expressed in rBCG.

The variable efficacy of BCG in humans has been extensively documented but is still not well understood. Differential sensitization from exposure to environmental mycobacteria is likely the key factor influencing the varying levels of BCG protection observed across populations. Lise Brandt et al. 2002 [174] have demonstrated that prior sensitization of mice with environmental mycobacteria can inhibit BCG multiplication and thereby prevent the induction of an efficient BCG-mediated immune response and protection against TB challenge [174]. Even though the precise mechanism has not been clarified, there is evidence that the inhibition of protective efficacy of BCG induced by environmental mycobacteria relates to the cross-reactivity of their antigens [175,176,177,178].

## 5. Safety of Recombinant BCG Vaccination in HIV-1-Infected Individuals

BCG vaccines are among the oldest vaccines and have been used in humans since 1921. They are the most widely used vaccine in the world, with over 3 billion doses delivered and a long-established, proven safety profile [179]. The available live-attenuated BCG vaccines are safe and effective, particularly to prevent the most severe forms of TB, such as childhood TB meningitis and miliary TB disease. They also provide protection against leprosy [180]. Furthermore, BCG vaccination at birth may have a non-specific beneficial effect and has been associated with lower child mortality and better responses to several major infections [181,182]. In addition, among the off-target effects of BCG vaccination, we should consider its immunomodulatory properties, which have sparked interest in its use as a preventive or therapeutic tool against allergic and autoimmune diseases [183]. However, safety issues of BCG vaccination have been raised among HIV- or HIV/TB infected populations. BCG vaccination is contraindicated in immunocompromised and HIV-infected individuals. Infants exposed to immunosuppressive treatments during pregnancy or breastfeeding should not receive the BCG vaccine administration. Importantly, children living with HIV who receive BCG at birth face a higher risk of developing disseminated BCG disease. However, HIV-infected individuals, including children, who are on antiretroviral therapy (ART), clinically stable, and immunologically healthy (CD4% > 25% for children aged 5 years) should be vaccinated with BCG. All in all, populations with a high prevalence of HIV infection also have the greatest burden of TB; in such populations, the benefits of potentially preventing severe TB through vaccination at birth are outweighed by the risks associated with the use of the BCG vaccine [180]. Daniel Faurholt-Jepsen et al. (2013) have shown, in a matched case-control study in Mwanza, Tanzania, that BCG vaccination considerably reduced the risk of TB, among individuals over the age of 15 both with and without HIV infection [184]. However, others clinical studies held in Angola and Zambia suggested that BCG does not have protective effect against TB among HIV-infected children [185,186].

Van der Meer et al. (2015) did postulate that mammalian innate immunity also exhibits an immunological memory, for which they propose the name of “trained immunity” [187,188]. They demonstrated that BCG vaccination in healthy volunteers resulted in a four- to seven-fold increase in IFN-γ production, along with a twofold rise in monocyte-derived cytokine release, including TNF and IL-1β, in response to unrelated bacterial and fungal pathogens. This enhanced monocyte function persisted for at least three months after vaccination and was associated with increased expression of activation markers such as CD11b and Toll-like receptor 4. They concluded that BCG induces trained immunity and non-specific protection from infections through epigenetic reprogramming of innate immune cells [189]. In addition, the same group showed that BCG vaccination induced trained immunity in NK cells, increasing the proinflammatory cytokine production in response to mycobacteria and unrelated pathogens in healthy volunteers [190].

Some preclinical studies in adult, newborn, and infant NHP model and BALB/c mice have shown that recombinant BCG or attenuated *Mtb* strains expressing several HIV/SIV antigens were safe and well tolerated [65,135,160,191]. However, despite these evident data, Jensen et al. (2017) [192] have shown that infant vaccinated monkeys with an attenuated recombinant *Mtb* strain expressing SIV Env and Gag required fewer SIV exposures to become infected compared to naive controls. They hypothesized that an imbalance between enhanced myeloid cell function and immune activation might have influenced the outcome of oral SIV challenge in AMtb-SIV-vaccinated infants [192]. Similarly, Seema M. Thayil et al. (2012) proposed that immune activation caused by *Mtb* or *M. bovis* BCG, the two most prevalent mycobacterial exposures globally, increases susceptibility to HIV infection [193]. They examined susceptibility to HIV infection in peripheral blood mononuclear cells (PBMC) stimulated with *Mtb* complex and identified enhanced susceptibility of CD4+ T cells to HIV infection through a TLR2- mediated pathway [193].

## 6. Concluding Remarks

Recombinant BCG expressing HIV antigens is a live-attenuated bacterial vaccine vector and is a promising approach for HIV vaccine development. Numerous studies have demonstrated that BCG:HIV is safe and can prime specific HIV-1 humoral and effector memory T-cell responses. Three key factors must be taken into account when developing a rBCG:HIV vaccine: (i) BCG codon optimization; (ii) antigen localization; and (iii) genetic plasmid stability in vivo. The BCG codon usage increases the mycobacterial transcriptional/translational activity of the heterologous gene. The secretion of antigens and fusion to mycobacterial surface lipoproteins increases MHC class I presentation, thereby enhancing immunogenicity. To prevent the disruption of heterologous gene expression in the *M. bovis* BCG host strain and improve genetic plasmid stability in vitro and in vivo, the use of integrative expression vectors, weak promoters, non-glycosylation sites in DNA fragments, small HIV DNA-coding sequences, and auxotrophic BCG mutant strains complemented by plasmids containing the complementing gene has been suggested and recommended.

Auxotrophic BCG strains replicate in vitro upon supplementation of the culture with specific amino acids but do not persist in vivo and the induced immunity may not be as long-lasting as that of conventional BCG. However, these strains would hopefully be completely safe in an immunocompromised host, including those at risk of HIV infection. Furthermore, as was discussed in this review, pre-immunization with BCG is unlikely to pose a significant limitation for the use of recombinant BCG vaccines in humans.

The specific- HIV-1 immune responses could be improved by: (i) using homologous or heterologous prime-boost regiments; (ii) assessing different BCG strains; (iii) fusing the HIV antigen to signal sequences peptides to facilitate the antigen secretion; (iv) changing the BCG strain; and (v) developing different strategies to overcome the antibody or T-cell immunodominance phenomenon.

BCG is associated with trained immunity and an increase in HIV-1 target cells. Thus, the immune activation assays should be incorporated to evaluate the vaccine safety profile. On the other hand, the low level of foreign antigen expression can be increased by using replicative multicopy expression vectors, inducible promoters, BCG codon usage, or strong promoters, or selecting different BCG strains. Finally, we believe that promising rBCG:HIV vaccines that have been shown to be immunogenic and protective in small animal models and non-human primates should be tested in human phase I clinical trials.
